# Expression of Pannexin 1 in the Human Kidney during Embryonal, Early Fetal and Postnatal Development and Its Prognostic Significance in Diabetic Nephropathy

**DOI:** 10.3390/biomedicines10050944

**Published:** 2022-04-20

**Authors:** Ivo Jeličić, Katarina Vukojević, Anita Racetin, Davor Čarić, Merica Glavina Durdov, Mirna Saraga-Babić, Natalija Filipović

**Affiliations:** 1 Renal Unit, University Hospital of Split, Šoltanska 1, 21000 Split, Croatia; ivo.jelicic@gmail.com; 2 Department of Anatomy, Histology and Embryology, Laboratory for Early Human Development, University of Split School of Medicine, Šoltanska 2, 21000 Split, Croatia; kvukojev@gmail.com (K.V.); anitamuic10@gmail.com (A.R.); msb@mefst.hr (M.S.-B.); 3 Department of Anatomy, Histology and Embryology, Laboratory for Neurocardiology, University of Split School of Medicine, Šoltanska 2, 21000 Split, Croatia; 4 Department of Orthopaedics and Traumatology, University Hospital of Split, Spinčićeva 1, 21000 Split, Croatia; caric.davor@gmail.com; 5 Department of Pathology, University of Split School of Medicine, Šoltanska 2, 21000 Split, Croatia; merigdst@yahoo.co.uk; 6 Department of Pathology, University Hospital of Split, Spinčićeva 1, 21000 Split, Croatia

**Keywords:** pannexin 1, human kidney, human embryo, early human development, diabetic nephropathy, chronic kidney disease

## Abstract

Pannexins are transmembrane glycoproteins that constitute channels involved in purinergic signaling through ATP release from cells in various physiological and pathological processes. In this study, the distribution of Panx1 expression in different cell populations of healthy postnatal human kidneys and during human embryonic and early fetal development was investigated by double immunohistochemistry. In addition, the glomerular and tubular expression of Panx1 was examined in patients with type 2 diabetes mellitus (DM2) and the control group, and renal Panx1 expression was correlated with serum creatinine. In the 6th week of embryonic development (DW), Panx1 expression was found in mesonephric glomeruli and mesonephric tubules. At the transition from 6th to 7th DW, Panx1 immunoreactivity was found in the mesonephric tubules and mesonephric duct, as well as in the metanephric ureteric bud and ampullae. In the 7th DW, strong Panx1 immunoreactivity was observed in the developing ureteric bud in the metanephros, whereas no Panx1 immunoreactivity was found in the metanephric cup. In the 8th DW, Panx1 expression was also found in the ureteric bud of the metanephros, the renal vesicle and comma-shaped nephron, and the epithelial cells of Bowman’s capsule. Expression of Panx1 was found at an early stage in both the paramesonephric duct and the mesonephric duct and diminished toward the 8th DW. During the 6th–10th DW, colocalization of Panx1 with alpha smooth actin (aSMA) was found in developing blood vessels. In the postnatal kidney, strong Panx1 immunoreactivity was present in medullary and cortical collecting duct cells, renin-producing cells, and proximal tubules. Very weak Panx1 immunoreactivity was found in certain distal tubule cells and the thin descending limbs of the loop of Henle. Panx1 immunoreactivity was also found in nephrin-immunoreactive podocytes. Panx1 was not colocalized with aSMA immunoreactivity in the vessels of the postnatal human kidney, but it was present in the endothelium. A significant positive correlation was found between Panx1 expression in glomeruli and serum creatinine only in diabetic patients and was not found in the nondiabetic group. The spatiotemporal expression of Panx1 during the early stages of human kidney development supports its possible role in cellular differentiation, migration, and positioning in the developing human kidney. In addition, our data suggest that glomerular Panx1 expression is a potential indicator of worsening renal function in patients with type 2 diabetes.

## 1. Introduction

Pannexins are transmembrane glycoproteins that constitute hexameric mechanosensitive and small-molecule permeable channels that play a role in paracrine communication between cells [1,2,3]. They are involved in purinergic signaling through ATP release from cells in various physiological and pathological situations [1,2]. Of the three members of the pannexin (Panx) family (Panx1, Panx2, and Panx3), Panx1 appears to be the most widely expressed. It has been found in various tissues and cell types, including the endothelium, epithelial cells, red blood cells, platelets, lymphocytes, adipocytes, muscle, brain, spleen, cartilage, skin, liver, kidney, bladder, lung, and colon [3,4].

The distribution of Panx1 was extensively studied in mouse kidneys. It was found to be present in the apical part of renal tubule cells, including proximal convoluted tubules, thin descending limbs and collecting ducts, the endothelium of renal vessels, smooth muscle cells of renal arteries, including afferent arterioles, and native or cultured podocytes [5,6,7,8]. Panx1 is thought to have several functions in the kidney, including the control of vascular function and fluid and electrolyte transport of the renal epithelium [5]. In addition, a role of Panx1 in the control of blood pressure has been suggested after a recent study documented the involvement of Panx1 in renin secretion [9]. Numerous studies have demonstrated that Panx1 mediates various pathological processes in addition to its physiological role. Pannexin channels play a role in inflammasome activation and the release of pro-inflammatory cytokines, leukocyte activation, and migration and are involved in various types of cell death (apoptosis, pyroptosis, autophagy) [4]. In particular, their role in the development of acute kidney injury (AKI) has been studied. Recently, it has been found that the upregulated caspase-11 promotes NLRP3 inflammasome activation via the cleavage of Panx1 in ischemia–reperfusion (IRI)-induced AKI [10]. Hence, the pharmacological targeting of Panx1 channels has been suggested to be protective against tubular injury in various AKI models in mice [7,11,12].

The complex development of the human urinary excretory organs includes three groups of successive structures: the pronephros, which appears in the fourth week and soon degenerates; the mesonephros, which begins to develop late in the 4th week and degenerates toward the end of the 12th week, and the primordia of the permanent kidney, the metanephros, whose development begins in the fifth week and becomes functional approximately in the 9th developmental week [11]. The exchange of the mesenchymal-to-epithelial transition (MET) [12] and a reverse process of the epithelial-to-mesenchymal transition (EMT) [13] play a key role in nephrogenesis, especially for podocyte development. Reciprocal induction is responsible for the initiation and regulation of MET, in which the ureteric bud (which arises from the mesonephric duct) and the metanephrogenic blastema (the metanephric mesenchyme) induce each other [11]. Molecular studies have shown that a complex interaction of different signaling systems is involved in these processes, including glial cell-derived neurotrophic factor (GDNF), Wingless-related integration site (Wnt) family, and bone morphogenetic protein (BMP) signaling pathways. Increasing evidence points to the role of intercellular communication via gap junctions and connexin hemichannels in kidney development [5]. We have recently described the expression of different connexin types in the human kidney during embryonic/early fetal development [14,15]. However, there are no data on the expression and role of Panx1 in embryonic and fetal kidney development in general and in the human embryo in particular.

Chronic kidney disease (CKD) is a growing health problem affecting approximately 10% of the world’s population. One of the main causes of CKD is diabetic nephropathy (DN), which is a leading cause of kidney failure (40% of new cases). Despite extensive studies, the pathophysiological mechanisms of DN are not completely understood, and new therapeutic approaches are needed. An increasing number of recent studies point to the role of direct cell-to-cell communication and paracrine ATP in the pathophysiology of DN, with connexin-formed channels playing the major role [16,17,18,19,20,21]. However, despite their central role in ATP-mediated signaling, a role for pannexins in DN has not yet been investigated. In our recent study of streptozotocin-induced diabetes mellitus in rats, we found almost exclusive expression of Panx1 in distal tubular cells from diabetic rats but not in those without diabetes [22]. Since we found that distal tubule cells were most severely damaged in the same diabetes model [23], we concluded that Panx1 might play a role in distal tubule damage during diabetes. To date, none of the studies have investigated this relationship in human kidneys.

Many pathological conditions are characterized by the activation of embryonic/fetal expression patterns of key regulatory molecules [24,25]. Cell–cell communication is essential for nephrogenesis, but also throughout the life span of the nephron, and its disruption leads to pathology [26,27]. Activation of developmental expression patterns may lead to the progression of damage in CKD, but may also play a role in repair and regeneration [27]. Therefore, the aim of this study was to compare Panx1 expression patterns during normal kidney development, in the normal postnatal kidney, and in the kidneys of patients with diabetes mellitus, a major cause of CKD. Overall, most of the data on the physiological and pathophysiological role of Panx1 in the kidney have been obtained by studying murine models. Even in rats, Panx1 has rarely been studied. There is only one study that demonstrated the expression of Panx1 in the human kidney, in which Panx1 was mentioned to be present mostly in tubular cells and its expression was increased in the renal tissue of patients with sepsis-related AKI [28]. However, details about the cellular distribution of Panx1 in the human kidney are not known. Moreover, the expression of Panx1 in the embryonic development of the human kidney has not been studied. In addition, there are no data on the expression of Panx1 in the kidneys of diabetic patients. Therefore, we used double immunohistochemistry to investigate the distribution of Panx1 expression in different cellular populations of a normal human kidney. We also examined the expression of Panx1 in the kidney during human embryonic and early fetal development. To explore the prognostic potential of Panx1 in diabetic nephropathy, we compared the glomerular and tubular expression of Panx1 between patients with type 2 diabetes mellitus and the control group and correlated renal Panx1 expression with serum creatinine as a potential indicator of renal function.

## 2. Materials and Methods

### 2.1. Tissue Procurement and Processing

Human conceptuses were obtained from the Department of Gynaecology and Obstetrics and the Department of Pathology after spontaneous abortions or ectopic pregnancies and processed with the approval of the Ethics and Medicines Committee of the Split University Hospital in accordance with the Declaration of Helsinki [29] (class: 003-08/16-03/0001, approval number: 2181-198-03-04-16-0024). The preservation of the material was checked and the poorly preserved material was discarded. The age of the conceptuses was estimated based on external measurements (crown–rump length) and Carnegie stages [30]. A total of 6 normal human conceptuses were collected between 5 and 10 weeks of development, and postnatal tissue was collected from the autopsy of a healthy 1.5-year-old child. The specimens were fixed in 4% paraformaldehyde in phosphate-buffered saline (PBS, pH 7.4).

We also analyzed the kidneys of 41 patients who had undergone nephrectomy due to renal carcinoma in the last three years at the University Hospital of Split, Croatia—21 patients with diabetes and 20 patients without diabetes. Healthy tissue adjacent to the carcinoma was separated and fixed by immersion in buffered 4% paraformaldehyde. Laboratory data at the time of nephrectomy were obtained from hospital records. Approval was obtained from the Ethics Committee of the Split Clinical Hospital (class: 500-03/21-01/158, approval number: 2181-147/01/06/M.S.-21-02).

### 2.2. Immunohistochemistry Procedure

Tissue and embryo specimens were washed several times in PBS, dehydrated in ethanol solutions, and embedded in paraffin wax, by using a standard procedure. Paraffin blocks were cut in the transversal plane (5 μm), and sections were mounted on glass slides.

Sections were then deparaffinized in xylene and rehydrated using a decreasing concentration solution of ethanol in water and rinsed with distilled water. Antigen retrieval was achieved by heating in sodium citrate buffer (pH 6.0) for 30 min in a steam cooker. Slides were then cooled down to room temperature, washed in PBS, and a protein block (ab64226, Abcam, Cambridge, UK) was applied and incubated for 20 min. The protein block was removed, and a combination of primary antibodies (Table 1) diluted in PBS was applied. The sections were incubated with primary antibodies overnight in a humid chamber. After washing in PBS, sections were incubated for 1 h in a humid chamber with an adequate combination of secondary antibodies (Table 1). Sections were then washed in PBS and nuclei were stained with 4′6′-diamidino-2-phenylindole dihydrochloride (DAPI). In case of double staining with lectins, the appropriate lectin (LTL or DBA) was applied before DAPI staining and incubated in a dark humid chamber at room temperature for 1 h, washed in PBS, and nuclei were stained with DAPI. After final washing with distilled water, the slides were cover-slipped (Immumount, Shandon, Pittsburgh, PA, USA). The exclusion of the primary antibody resulted in no staining in the tissue.

### 2.3. Data Acquisition and Analysis

Stained sections were viewed and photographed using a BX51 microscope (Olympus, Tokyo, Japan) equipped with a cooled digital camera (DS-Ri2; Nikon, Tokyo, Japan) using NIS-Elements F software. The used objectives were: UPLFLN4X, UPLFLN10X2, UPLFLN40X, and UPLFLN100XO2 (all Olympus, Tokyo, Japan). Green granular deposits were interpreted as positive Panx1 immunoexpression, except in the case of colocalization with lectins (LTL and DBA) and renin, where red granular deposits were interpreted as positive Panx1 immunoexpression. In order to quantify Panx immunoexpression in diabetic and nondiabetic specimens’ glomeruli, visual fields captured at an objective magnification of 40× and constant exposure time were analyzed, and for tubule/interstitial compartment analysis, the x20 objective was used to capture 10 non-overlapping fields that contained cortical tubules. Each field formed one image.

Photomicrographs were processed and analyzed using ImageJ software (National Institutes of Health, Bethesda, MD, USA). The red counter-signal for green fluorescence was subtracted from the images with green staining to reduce fluorescence leakage. The median filter with a radius of 5.0 pixels for the glomeruli and 7.0 pixels for the tubule/interstitial area was applied and pictures were adjusted to the threshold method with a “Triangle” thresholding algorithm. The area’s fluorescence percentage was determined using the Analyze Particles function. For the analysis of glomeruli, particular structures were manually outlined and isolated by using Adobe Photoshop (Adobe Inc., San Jose, CA, USA), while the entire picture area was used for analysis of the tubule/interstitial compartment. Then, photographs were processed as described above and the percentage area fraction in glomeruli/tubules was calculated. For the purpose of presentation, subtraction of background and contrasting was performed.

### 2.4. Statistical Analysis

Normality of data distribution was assessed using the Kolmogorov–Smirnov test. The Mann–Whitney test was used to compare differences between patient groups. The Spearman correlation test was used to determine correlations between variables. Using the Statistical Package for Social Science (SPSS, version 23.0), calculations were performed. A *p*-value of less than 0.05 was considered significant.

## 3. Results

### 3.1. Renal Expression of Panx1 in the Human Kidney during Embryonic and Early Fetal Development

#### 3.1.1. Sixth to Seventh Week of Development

**Figure 1:**
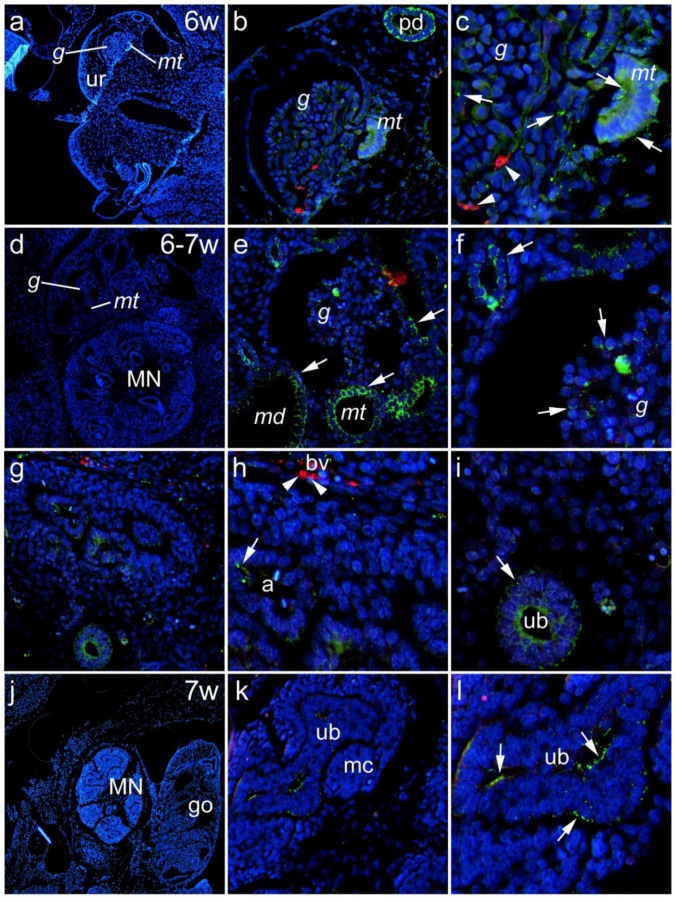
Expression of Panx1 during the 6th to 7th week of human renal development. During the 6th week of development, mesonephric structures (glomeruli—g, mesonephric tubules—mt) could be seen in the urogenital ridge—ur (**a**). Panx1 expression was found in mesonephric glomeruli—g and mesonephric tubules—mt (arrows), while aSMA-positive cells were seen in the blood vessels walls within the glomeruli. Moreover, strong Panx1 immunoreactivity was found in a paramesonephric duct (pd); magnification of panel a (**b**). Details of Panx1 and aSMA positivity (arrowheads) can be seen on the magnification of panel b (**c**). In the transition from 6th to 7th embryonal week, both mesonephric structures (glomeruli—g, mesonephric tubules—mt) and metanephric (MN) structures could be seen in close proximity (**d**). Strong Panx1 immunoreactivity (arrows) was found in mesonephric tubules (mt) and the mesonephric duct—md; magnification of panel d (**e**) and panel e (**f**). Panx1-positive cells (arrows) could be seen in the metanephros ureteric bud (ub) ampullae (a), whereas aSMA-positive cells (arrowhead) were seen in the blood vessels—bv (**g**). Magnification of panel g (**h**). Magnification of ureteric bud (ub) from panel g (**i**). At the 7th week of development, the metanephros and gonad (go) could be seen (**j**). From metanephric structures, strong Panx1 immunoreactivity (arrows) was observed in the developing ureteric bud (ub), while the metanephric cup (mc) was negative (**k**). Magnification of panel k (**l**).

We examined the renal expression of Panx1 during the embryonic and early fetal periods. During the 6th week of development, mesonephric structures (glomeruli, mesonephric tubules) could be seen in the urogenital ridge (Figure 1a). Panx1 expression was found in mesonephric glomeruli and mesonephric tubules, whereas aSMA-positive cells were seen in blood vessel walls within glomeruli. In addition, strong Panx1 immunoreactivity was found in a paramesonephric duct (Figure 1b). Panx1 immunofluorescence was present in aSMA-immunoreactive cells (Figure 1c). In the transition from the 6th to the 7th embryonic week, both mesonephric structures (glomeruli and mesonephric duct) and metanephric structures were seen in close proximity (Figure 1d). Strong Panx1 immunoreactivity was found in the mesonephric tubules and the mesonephric duct (Figure 1d–f). Panx1 immunoreactive cells were seen in the ureteric bud of the metanephros and in the ampullae, whereas aSMA-positive cells were found in the blood vessels (Figure 1g–i). In the 7th week of development in metanephric structures, strong Panx1 immunoreactivity was observed in the developing ureteric bud, while in the metanephric cup, Panx1 immunoreactivity was not found (Figure 1k,l).

#### 3.1.2. Eighth to Tenth Week of Development

**Figure 2:**
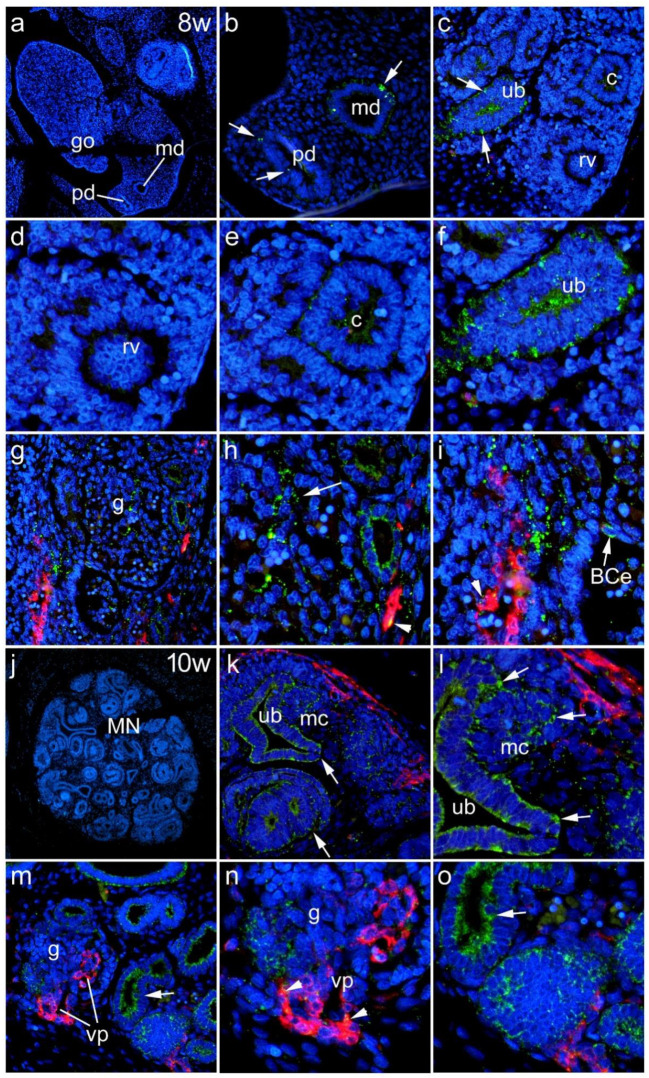
Expression of Panx1 during the 8th to 10th week of human kidney development. In the 8th developmental week, paramesonephric duct (pd) and mesonephric duct (md) were seen on the lateral side of the developing gonad (go) (**a**). Expression of Panx1 was present in both the paramesonephric duct (pd) and the mesonephric duct (md) (**b**). In addition, Panx1 expression was also found in the ureteric bud (ub) of the metanephros (**c**). In addition, Panx1 immunoreactivity was not detected in the renal vesicle—rv (**d**), but was detected in the comma-shaped nephron—c (**e**) and ureteric bud (**f**). Panx1 immunoreactivity (arrows) was observed in the epithelial cells of Bowman’s capsule—BCe, and they also colocalized with aSMA (arrowheads) in the developing vessels of the vascular glomerular pole (vp) and in the extraglomerular vessels (**g**–**i**). At week 10 of development (**j**), strong Panx1 immunoreactivity was found in all tubules and in aSMA-immunoreactive vessels of the vascular glomerular pole and extraglomerular vessels (**k**–**o**); MN—metanephros; mc—metanephric cup; g—glomerulus.

At week 8 of development, the paramesonephric duct and the mesonephric duct could be seen on the lateral side of the developing gonad (Figure 2a). Expression of Panx1 was present in both the paramesonephric and mesonephric ducts (Figure 2b), although to a lesser extent than in earlier developmental stages (Figure 2b). In addition, Panx1 expression was also found in the ureteric bud of the metanephros (Figure 2c). Moreover, Panx1 immunoreactivity was rare in the renal vesicle (Figure 2d), present in the comma-shaped nephron (Figure 2e), and significantly lower compared with the ureteric bud (Figure 2f).

**Figure 3:**
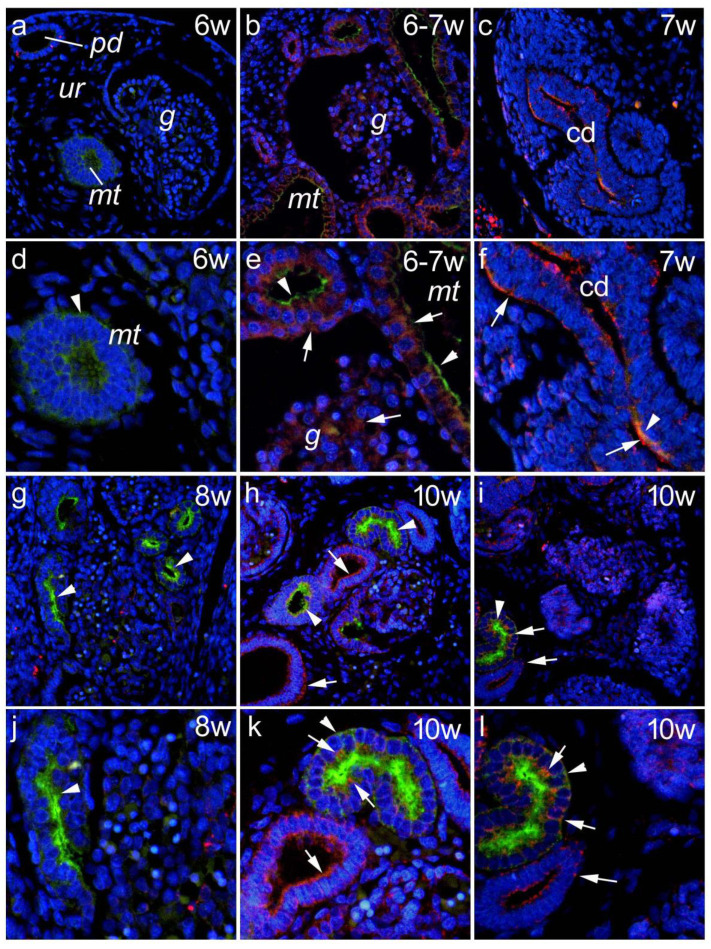
*Lotus tetragonolobus* lectin (LTL) binding during embryonic and early fetal development and its colocalization with Panx1 expression. During the 6th week of development, mesonephric structures (glomeruli—g, mesonephric tubules—mt) were seen in the urogenital ridge—ur; weak LTL binding (green; arrowheads) was observed in the mt; Panx1 (red) was positive in the paramesonephric duct (pd) (**a**). At the 6–7 w, Panx1 was expressed in mesonephric glomeruli—g and mesonephric tubules—mt (arrows), whereas LTL binding (arrowheads) was observed in mt (**b**). At the 7th week, in metanephros, Panx1 was strongly positive in collecting ducts (cd) (**c**). Magnification of panel a (**d**). Magnification of panel b (**e**). Magnification of panel c (**f**). Stronger and more consistent LTL immunofluorescence was found at later stages (8th–10th DW)—arrowheads—and it colocalized with Panx1 expression at 7th and 10th DW—arrows (**g**–**i**). Magnification of panel g (**j**). Magnification of panel h (**k**). Magnification of panel i (**l**).

Panx1 immunoreactivity was observed in the epithelial cells of Bowman’s capsule. In addition, we found colocalization of Panx1 with aSMA in the developing vessels of the glomerular vascular pole as well as in extraglomerular vessels (Figure 2g–i). During the 8th week, Panx1 expression was more pronounced in *Lotus tetragonolobus* lectin (LTL)-negative tubules, and during this period, it appeared to diminish from LTL-positive tubules (Figure 3). During the early fetal period, at week 10 of development, strong Panx1 immunoreactivity was found in all LTL-positive and -negative tubules and in the aSMA-immunoreactive vessels of the vascular glomerular pole and extraglomerular vessels (Figure 2k–o and Figure 3).

#### 3.1.3. *Lotus Tetragonolobus* Lectin Binding during Embryonic and Early Fetal Development and Its Colocalization of Panx1 Expression

Weak LTL binding, a marker for proximal tubules, was observed as early as the 6th week of development for mesonephric structures in mesonephric tubules (Figure 3a,d). Panx1 immunoreactivity was also found in the paramesonephric duct (Figure 3a).

At weeks 6–7, LTL binding was observed in the mesonephric tubules, where it colocalized with Panx1, whose expression was also found in the mesonephric glomeruli (Figure 3b,e). At week 7, Panx1 was strongly positive in the metanephric collecting ducts, where it colocalized with intense LTL immunofluorescence (Figure 3c,f). Stronger and more consistent LTL immunofluorescence was found at the later stages (8th to 10th DW), and it colocalized with Panx1 expression at 7th and 10th DW (Figure 3g–l).

### 3.2. Renal Expression of Panx1 in Postnatal Human Kidney

**Figure 4:**
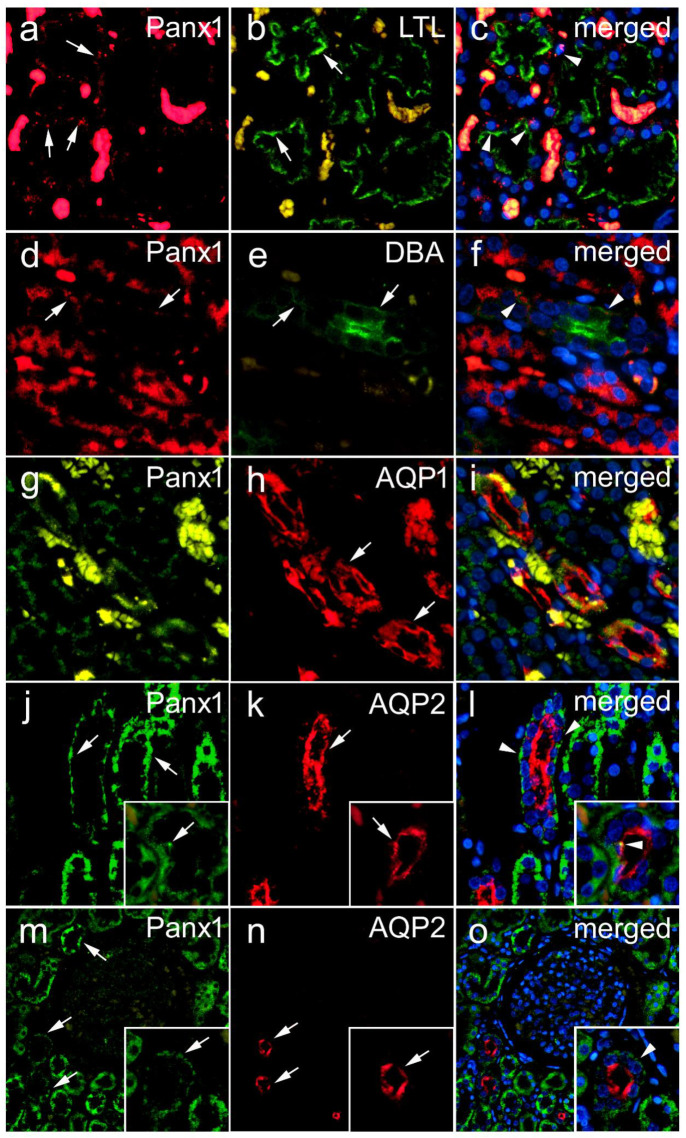
Expression of Panx1 in different tubular segments of postnatal human kidney. Double immunohistochemistry was used to localize the expression of Panx1 in different nephron segments. Left panel—Panx1 (**a**,**d**,**g**,**j**,**m**). Middle panel—tubular segment markers (**b**,**e**,**h**,**k**,**n**): LTL—*Lotus tetragonolobus* lectin, a marker for proximal tubules (**b**); DBA—*Dolichos biflorus* agglutinin, a marker for distal tubules (**e**); AQP1—Aquaporin 1, a marker for a thin descending limb of the loop of Henly (**h**); AQP2—Aquaporin 2, a marker for collecting ducts (in medulla and cortical collecting ducts) (**k**,**n**). Right panel—both (Panx1 and specific marker) merged with nuclear DAPI staining (**c**,**f**,**i**,**l**,**o**). Arrow—positive immunoexpression; arrowheads—colocalization.

**Figure 5:**
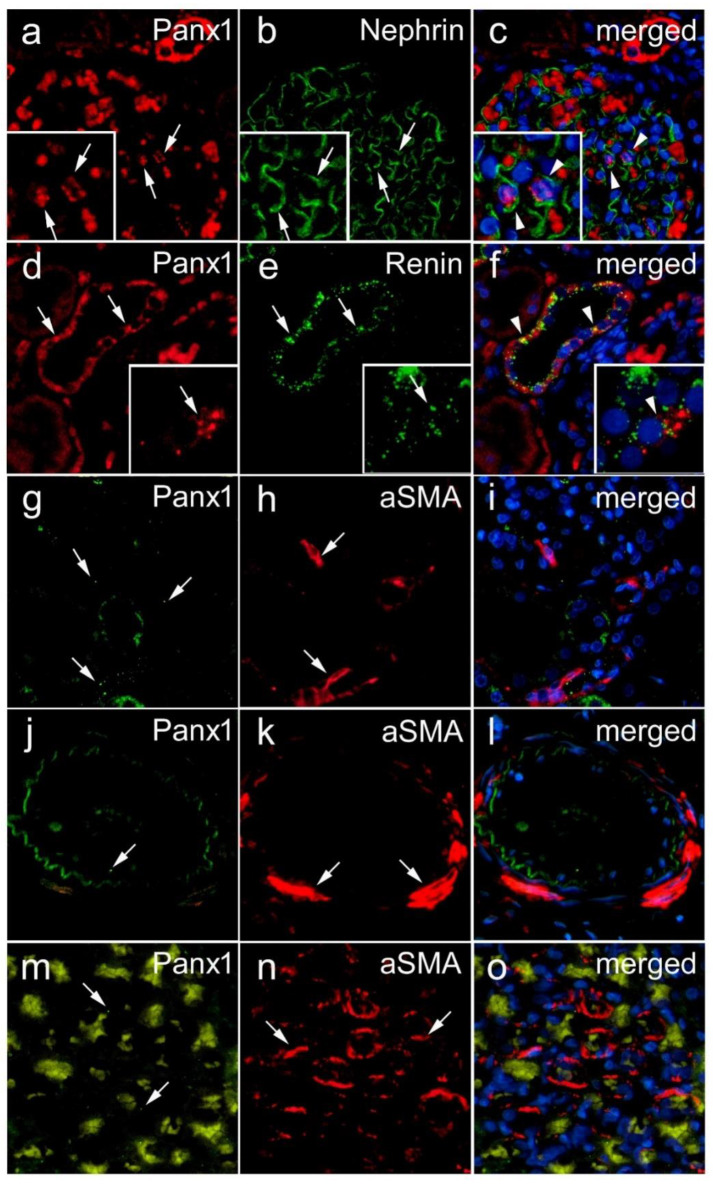
Glomerular and vascular expression of Panx1 in postnatal human kidney. Double immunohistochemistry was used to localize Panx1 expression in podocytes, renin-producing cells, and vascular smooth muscle cells. Left panel—Panx1 (**a**,**d**,**g**,**j**,**m**). Nephrin—a marker for podocytes; aSMA—alpha-smooth actin, a marker for vascular smooth muscle cells (here in the vessel wall). Middle panel—specific marker for podocytes, renin-producing cells, or smooth muscle cells (**b**,**e**,**h**,**k**,**n**). Right panel—both (Panx1 and specific marker) merged with nuclear DAPI staining (**c**,**f**, **i**,**l**,**o**). Arrow—positive immunoexpression; arrowheads—colocalization.

In the renal cortex, the most intense Panx1 immunoreactivity was observed in tubular structures. Although in lower density, Panx1 immunoreactive puncta were also seen in glomeruli. In different parts of the renal medulla, the intensity of Panx1 immunoreactivity varied from very strong to weak depending on the type of tubular structures, and it appeared to be absent in the muscular layer of the blood vessels. By a double immunohistochemical colocalization study, we confirmed the existence of Panx1 expression in different populations of renal cells in the postnatal human kidney (Figure 4 and Figure 5). Colocalization with LTL binding revealed the expression of Panx1 in the proximal tubules of the human kidney (Figure 4a–c). Although weak, Panx1 immunoreactivity was also found in certain distal tubule cells, as shown by colocalization with *Dolichos biflorus* lectin binding (Figure 4d–f). In addition, co-labeling with aquaporin 1 (AQP1) revealed the expression of Panx1 in the thin descending limbs of the loop of Henle (Figure 4g–i). However, the most intense Panx1 expression in the medulla was found in the cells of the collecting ducts, as shown by colocalization with aquaporin 2 (AQP2) immunoreactivity (Figure 4j–l). In addition, Panx1 immunoreactivity colocalized with AQP2 was also found in the cortical collecting ducts (Figure 4m–o). In postnatal glomeruli, we found Panx1 immunoreactivity in nephrin-immunoreactive podocytes (Figure 5a–c), although it was much less pronounced than its expression in tubular structures. In addition, we found strong Panx1 expression in renin-immunoreactive cells (Figure 5d–f). However, in contrast to the embryonic and early fetal periods, we did not find colocalization between Panx1 and aSMA immunoreactivity in the postnatal human kidney, either in cortical vessels in the vascular glomerular pole (Figure 5g–i), large arteries (Figure 5j–l), or medullary vessels (Figure 5m–o). Rather, Panx1 immunoreactivity was sometimes present in the endothelium (Figure 5g–o).

### 3.3. Expression of Panx1 in Diabetic and Nondiabetic Renal Specimens

**Figure 6:**
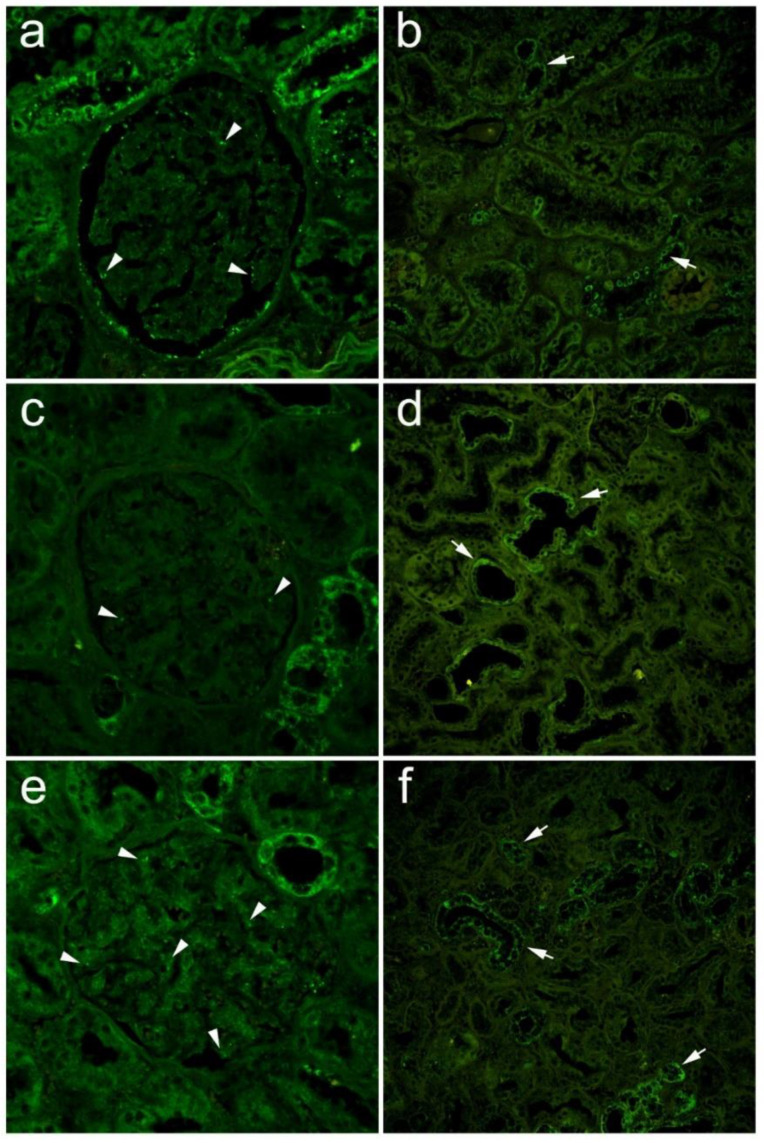
Expression of pannexin 1 in glomeruli and tubules of nondiabetic and diabetic patients. Renal sections were stained immunohistochemically by using anti-Panx1 antibody (green). (**a**) Glomerulus of nondiabetic patient with normal creatinine; (**b**) tubuli of the same patient; (**c**) glomerulus of diabetic patient with normal creatinine; (**d**) tubuli of the same patient; (**e**) glomerulus of diabetic patient with elevated creatinine; (**f**) tubuli of the same patient. Arrowheads—Panx1 expression in glomeruli; arrows—Panx1 expression in tubuli.

Kidney samples from 41 patients (29 men and 12 women) were analyzed (Table 2). Twenty-one of them were confirmed to have diabetes, while 20 were not diabetic. The mean age of the patients was 66.7 ± 9.3 years (44–89). Representative photomicrographs are presented in Figure 6. Preoperative serum creatinine was 103.4 ± 42.2 μmol/L (50–250 μmol/L). In the diabetic group, there were 13 men and 8 women; the mean age was 68.6 ± 9.9 years (49–89) with a serum creatinine of 107.3 ± 54.2 μmol/L (61–250 μmol/L). In the nondiabetic group, there were 14 men and 5 women, the mean age was 64.4 ± 8.8 years (44–75), and the mean preoperative serum creatinine was 99.6 ± 27.8 μmol/L (50–164 mol/L). We found no statistically significant difference between groups in serum creatinine (*p* = 0.66) or age (*p* = 0.202).

Four hundred and ten glomeruli were analyzed (210 from diabetic and 200 from nondiabetic patients). Panx1 expression was higher in the glomeruli (9.45 ± 8.13% vs. 7.57 ± 5.13%) and tubular/interstitial (2.82 ± 1.3% vs. 2.5 ± 0.99%) of diabetics, but without statistical significance (*p* = 0.828 and *p* = 0.056, respectively) (Table 2). We found a statistically significant positive correlation between Panx1 expression in glomeruli and serum creatinine (rho = 0.162, *p* = 0.002) and patient age (rho = 0.151, *p* = 0.003) (Table 3). In a subgroup of patients with diabetes, this correlation with creatinine (rho = 0.265, *p* < 0.001) and age (rho = 0.227, *p* < 0.001) was even more pronounced. In nondiabetic patients, there was no statistically significant correlation between Panx1 expression in glomeruli and serum creatinine (*p* = 0.914) or with age (*p* = 0.384).

On the other hand, we found no statistically significant correlation between tubular/interstitial Panx1 expression and serum creatinine (*p* = 0.543), but there was a statistically significant positive correlation between tubular/interstitial Panx1 expression and age (rho = 0.105, *p* = 0.044). In a subgroup of patients with diabetes, no correlation was found with creatinine (*p* = 0.963), but there was a statistically significant positive correlation with age (rho = 0.162, *p* = 0.003), which was even more pronounced. In nondiabetic patients, we found no statistically significant correlation between Panx1 expression and serum creatinine (*p* = 0.344) or with age (*p* = 0.089).

## 4. Discussion

Intercellular communication via gap junctions and paracrine ATP signaling pathways plays an important role in the normal morphogenesis and function of various organ systems [31,32,33]. Despite intensive research on the role of intercellular communication via connexin-constituted channels in kidney development, there are no data on the expression and role of Panx1 in embryonic and fetal development, especially in the human embryo. Previous studies have shown that the spatiotemporal change in the expression of cell proliferation and apoptosis markers, some intermediate filaments, and growth factors during normal renal development is stage-dependent (renal vesicle, S-shaped body, capillary loop stage, renal corpuscle) [25,34,35,36]. Therefore, the aim of this study was to investigate the spatiotemporal Panx1 expression in human kidneys during embryonic and early fetal development.

During the 6th week of embryonic development, we found Panx1 expression in mesonephric glomeruli and mesonephric tubules. In the transition from the 6th to the 7th week of embryonic development, strong Panx1 immunoreactivity was found in mesonephric tubules and the mesonephric duct. In addition, Panx1 immunoreactive cells could be seen in the metanephric ureteric bud and ampullae. At week 7 of development in the metanephros, strong Panx1 immunoreactivity was observed in the developing ureteric bud, while in the metanephric cup, Panx1 immunoreactivity was not found. The presence of Panx1 in the ureteric bud derived from the mesonephric duct suggests that the ureteric bud might also use purinergic signals to induce the metanephrogenic mesenchyme to undergo nephrogenesis (formation of the metanephric vesicle). In support of this theory, Hillman and coworkers [37] discovered in developing mouse kidneys that one of the purinergic signaling receptors—P2X7—was expressed at day E13 in the condensing mesenchyme adjacent to the ureteric bud, indicating that the condensing mesenchyme of the metanephric cup is capable of responding to purinergic signals. In contrast, they found that ureteric bud branches, S-shaped bodies, primitive glomeruli, and proximal tubules did not express P2X7. According to our finding of Panx1 in the ureteric bud, the ureteric bud is able to release the purinergic signal via Panx channels, which then subsequently might bind to P2X7 receptors in the metanephric cup mesenchyme. At week 8 of development, Panx1 expression was also found in the ureteric bud of the metanephros, as well as in the renal vesicle and comma-shaped nephron. In addition, Panx1 immunoreactivity was observed in the epithelial cells of Bowman’s capsule. Consistent with previous findings, it could be suggested that purinergic signaling in the ureteric bud may be important for the epithelial–mesenchymal transition during the induction of the renal vesicle. Later, when the epithelial phenotype of metanephric tubule cells is reached, they also begin to express Panx1 and could potentially contribute to purinergic signaling.

The expression of Panx1 that we found at an early stage in both the paramesonephric duct and the mesonephric duct, which diminished toward the 8th developmental week, may indicate the importance of Panx1 in the early development of the paramesonephric and mesonephric duct, which diminishes toward the 8th week of development.

The early appearance of LTL binding found in the metanephric tubules and the gradual increase in fluorescence intensity with developmental age indicate the early commitment of tubular cells to achieve the phenotype of the specific tubular segment. However, from the LTL-Panx1 double staining results, we concluded that Panx1 expression during early embryonic kidney development was not restricted to a specific tubular segment.

During the 6th week, aSMA-positive cells were already seen in the blood vessel walls within the glomeruli, and Panx1 immunofluorescence was present in aSMA-immunoreactive cells. At week 8 of development, colocalization of Panx1 with aSMA was found in the developing vessels of the vascular glomerular pole and extraglomerular vessels, and it was even more accentuated during the early stages of the fetal period, at week 10 of development. These results indicate the important role of Panx1 in the development of the vascular structures of the glomerular vascular pole and extraglomerular renal vessels during early fetal development.

The cellular localization of Panx1 in the human kidney is not known and there are limited studies in rodent kidneys [7,38]. Therefore, in the present study, we used a double immunohistochemical technique to determine the localization of Panx1 in different cellular populations of the human kidney. We found the most intense Panx1 immunoreactivity in the tubular structures of the renal cortex in the postnatal human kidney. Although at lower density, Panx1 immunoreactive puncta were also seen in glomeruli. In different parts of the renal medulla, the intensity of Panx1 immunofluorescence varied from very strong to weak, depending on the type of tubular structure, and it appeared to be absent in the muscle layer of blood vessels. A double immunohistochemical colocalization study allowed us to confirm the existence of Panx1 expression in different populations of renal cells in the postnatal human kidney. The most intense Panx1 immunoreactivity was present in the cells of collecting ducts in the medulla and cortical collecting ducts. Colocalization with LTL binding revealed the expression of Panx1 in the proximal tubules of the human kidney. Very weak Panx1 immunoreactivity was also found in certain distal tubule cells (DBA-immunoreactive) and the thin descending limbs of the loop of Henle (AQP1-immunoreactive). These findings are in partial agreement with the results of a study in mice [5], where strong Panx1 immunoreactivity was found in cortical and medullary tubule segments. In particular, Panx1 was detected in the proximal tubule, the thin descending segment of the loop of Henle, and the collecting duct system. The main difference between our study in the human kidney and the results of Hanner et al. [5] on the mouse kidney is that we found only rare Panx1 immunoreactivity in a thin descending limb of the loop of Henley, according to colocalization with the AQP1 marker. The presence of Panx1 in the tubular epithelium is likely related to the release of ATP into the tubular lumen, which has previously been shown to be Panx1-dependent in mice, and it has been suggested that Panx1 may be involved in the control of fluid and electrolyte transport of the renal epithelium [5,7].

Panx1 immunoreactivity was also found in nephrin-immunoreactive podocytes. Our finding of Panx1 in glomeruli and its presence in nephrin-immunoreactive podocytes of the postnatal human kidney agrees with the previous results of Li and collaborators [8], who found abundant expression of Panx1 in cultured and native murine podocytes. Their study revealed that Panx1 channels can mediate anion conduction across the plasma membrane of podocytes, allowing the transport of ATP.

The strong Panx1 immunoreactivity that we found in renin immunoreactive cells is consistent with previous findings and supports the important role of Panx1 in the intercellular communication of granular cells during blood pressure regulation [9]. However, the lack of any colocalization between Panx1 and aSMA immunoreactivity in the vasculature of the postnatal human kidney, in contrast to what we found in the embryonic and early fetal periods, is not consistent with findings in mouse kidneys [5], where strong Panx1 immunoreactivity was found in aSMA-positive vascular smooth muscle cells. This may indicate species variability and a more important role of Panx1 in smooth muscle cell communication during intrauterine development in humans. The Panx1 immunoreactivity that we found in the endothelium is consistent with previous findings [6] in the mouse arterial network and supports the important role of Panx1 in the intercellular communication of vascular endothelial cells. Indeed, Lohman and coworkers [6] found that Panx1 was the only Panx consistently expressed in the endothelium throughout the arterial tree, regardless of artery size. They also found that Panx1 expression in smooth muscle cells was barely detectable in the larger conduit arteries but demonstrated some expression of Panx1 in smooth muscle cells in renal arteries and large and small coronary arteries. However, in the aforementioned study, no colocalization with aSMA, a smooth muscle cell marker, was performed, which may lead to a somewhat different interpretation of the results compared with our study.

Chronic kidney disease, as the main cause of renal failure, often a consequence of diabetic nephropathy (DN), is a growing global health problem that requires new diagnostic and therapeutic approaches based on better knowledge of the pathophysiological mechanisms. An increasing number of studies point to direct cell-to-cell communication and a paracrine ATP role in the pathophysiology of DN [16,17,18,19,20,21]. Despite extensive studies on the role of purinergic signaling in various renal pathologies [26], the role of Panx1, one of the major channels contributing to extracellular ATP release, has not been investigated in these processes. In our study, we found a significant positive correlation between Panx1 expression in glomeruli and serum creatinine, which was even more pronounced in a subgroup of patients with diabetes and was not found in the nondiabetic group. This correlation of glomerular Panx1 expression with the severity of renal damage could also be explained by the conclusions of the study mentioned earlier, according to which the physiological tonic inhibition of Panx1 channel activity may be attenuated under pathological conditions—for example, in obesity due to hypoadiponectinemia [8]. Under such conditions, increased Panx1 activity will lead to ATP release, inflammasome activation, and a consequent inflammatory response [4]. A role for Panx1 channels in renal pathology is also supported by the findings that the pharmacological inhibition of Panx1 by carbenoxolone, as well as the genetic deletion of Panx1, attenuates renal IRI in mice [7]. In the same study, the genetic deletion of Panx1 in proximal tubules or vascular endothelial cells was found to protect mice from renal IRI. However, the aforementioned study did not investigate the impact of Panx1 deletion in other renal cell populations, including podocytes or mesangial cells [7,38]. However, the authors suggested Panx1 as a potential pharmacological target in AKI and proposed several mechanisms of its renoprotective effect in the Panx1-deficient mice, including reduced inflammation with decreased cytokine and adhesion molecule expression and neutrophil infiltration [7]. In addition, a recent study confirmed the role of Panx1 cleavage by caspase-11 in NLRP3 inflammasome activation during IRI-caused AKI, connecting the Panx1 role with pro-apoptotic processes [10]. The detailed mechanism of Panx1 deletion effects in renal IRI might be explained by the results of the recent study conducted by Kirby and collaborators [39], who found that Panx1 channels are involved in the control of the hemodynamic response to hypoxia by regulating extracellular ATP in the blood.

In contrast to AKI, in which renal damage originates mainly from the proximal tubules, pathological processes in CKD usually begin in the glomeruli. Diabetic nephropathy, one of the main causes of CKD, usually begins with glomerular hyperfiltration and hypertrophy and continues as inflammation in the tubule/interstitial compartment. Moreover, the accumulation of extracellular matrix proteins leads to progressive glomerulosclerosis and tubulointerstitial fibrosis [40,41]. However, AKI is one of the diabetic complications and may lead to CKD if left untreated [38]. The results of our study showed no difference in the expression of Panx1 in the glomeruli or tubule/interstitial compartment between the diabetic and control groups of patients. However, the correlation between the severity of the renal injury, as measured by serum creatinine, and glomerular Panx1 expression observed in the diabetic patient group suggests the prognostic value of glomerular Panx1 expression and the potential of pharmacological Panx1 modulation in the treatment of DM-induced CKD. Although several pharmacological agents already approved by the FDA for other diseases are known to inhibit Panx1 channels [38], further research is needed to find more selective Panx1 inhibitors and to investigate whether Panx1 inhibition can reduce renal damage in CKD and their potential for the treatment of CKD.

## 5. Conclusions

To the best of our knowledge, this is the first study on the distribution of Panx1 in human kidney tissue. The presented data give us new information about the expression of Panx1 in different cells of the human kidney, and its dynamics during embryonic and early fetal development. The spatiotemporal expression pattern of Panx1 during the early stages of human kidney development supports its possible role in processes of cellular differentiation, migration, and positioning in the developing human kidney. In addition, our data suggest that glomerular Panx1 expression is a potential indicator of worsening renal function in patients with type 2 diabetes.

## Figures and Tables

**Table 1:** Primary and secondary antibodies used.

	Antibody	Code No.	Host	Dilution	Source
Primary	Anti-Pannexin 1/PANX1	ABN242	Rabbit	1:300	Merck KGaA, Darmstadt, Germany
	Anti-Nephrin (B-12)	sc-377246	Mouse	1:50	Santa Cruz Biotechnology Inc., Santa Cruz, CA, USA
	Anti-Aquaporin 1/AQP1 (B-11)	sc-25287	Mouse	1:50	Santa Cruz Biotechnology Inc., Santa Cruz, CA, USA
	Anti-Aquaporin 2/AQP2 (E-2)	sc-515770	Mouse	1:50	Santa Cruz Biotechnology Inc., Santa Cruz, CA, USA
	Anti-Renin [7D3-E3]	ab134783	Mouse	1:50	Abcam, Cambridge, UK
	Anti-Smooth Muscle Actin	M0851	Mouse	1:300	Dako, Glostrup, Denmark
Lectins	Fluorescein labeled *Dolichos biflorus* agglutinin (DBA)	FL-1031	-	1:400	Vector Laboratories Ltd., Peterborough, UK
	Fluorescein labeled Lotus Tetragonolobus lectin (LTL)	FL-1321	-	1:400	Vector Laboratories Ltd., Peterborough, UK
Secondary	Alexa Fluor^®^488 AffiniPure Anti-Mouse lgG (H+L)	715-545-150	Donkey	1:400	Jackson Immuno Research Laboratories, Inc., Baltimore, PA, USA
	Alexa Fluor^®^488 AffiniPure Anti-Rabbit lgG (H+L)	711-545-152	Donkey	1:400	Jackson Immuno Research Laboratories, Inc., Baltimore, PA, USA
	Rhodamine Red™-X (RRX) AffiniPure Anti-Mouse IgG (H+L)	715-295-151	Donkey	1:400	Jackson Immuno Research Laboratories, Inc., Baltimore, PA, USA
	Rhodamine Red™-X (RRX) AffiniPure Donkey Anti-Rabbit IgG (H+L)	711-295-152	Donkey	1:400	Jackson Immuno Research Laboratories, Inc., Baltimore, PA, USA

**Table 2:** Patient age, sex, serum creatinine, and expression of Panx1 in glomeruli and tubules.

					Panx 1	Panx 1
	N	N	Age	Creatinine	Glomeruli	t/i
	Male	Female	(Years)	(μmol/L)	% Area	% Area
All	29	12	66.7 ± 9.43 (44–89)	103.4 ± 42.2 (50–250)	8.53 ± 6.89 (1.94–41.33)	2.67 ± 1.16 (0.85–1.13)
Nondiabetic	16	4	64.4 ± 8.8 (44–75)	99.6 ± 27.8 (50–164)	7.57 ± 5.13 (2.01–34.2)	2.5 ± 0.99 (0.85–8.13)
Diabetic	13	8	68.6 ± 9.9 (49–89)	107.3 ± 54.2 (61–250)	9.45 ± 8.13 (1.94–41.33)	2.82 ± 1.3 (0.9–6.54)

**Table 3:** Spearman’s coefficient of correlation of Panx1 expression in glomeruli and tubules with patient age and serum creatinine.

		Panx 1 % Area Glomeruli	Panx 1 % Area t/i
All	Age	0.151	0.105
		***p* = 0.003**	***p* = 0.044**
	Creatinine	0.162	−0.033
		***p* = 0.002**	*p* = 0.543
Nondiabetic	Age	0.065	0.089
		*p* = 0.384	*p* = 0.249
	Creatinine	0.008	−0.071
		*p* = 0.914	*p* = 0.344
Diabetic	Age	0.227	0.213
		***p* < 0.001**	***p* = 0.003**
	Creatinine	0.265	0.004
		***p* < 0.001**	*p* = 0.963

Significant correlations are presented in bold.

## Data Availability

Data related to this study are available upon reasonable request from the corresponding author.
